# Comparative iTRAQ proteomics identified proteins associated with sperm maturation between yak and cattleyak epididymis

**DOI:** 10.1186/s12917-021-02907-9

**Published:** 2021-07-26

**Authors:** Wangsheng Zhao, Siraj Ahmed, Junxia Liu, Saeed Ahmed, Eugene Quansah, Tajmal Hussain Solangi, Yitao Wu, Yueling Yangliu, Hongmei Wang, Jiangjiang Zhu, Xin Cai

**Affiliations:** 1 School of Life Science and Engineering, Southwest University of Science and Technology, Mianyang, 621010 Sichuan China; 2 Qingdao Bright Moon Seaweed Group Co., ltd, Qingdao, 266400 Shandong China; 3 Key Laboratory of Qinghai-Tibetan Plateau Animal Genetic Resource Reservation and Utilization (Southwest Minzu University), Ministry of Education, Chengdu, 610041 Sichuan China; 4 Qinghai-Tibetan Plateau Animal Genetic Resource Reservation and Utilization Key Laboratory of Sichuan Province, Chengdu, 610041 Sichuan China

**Keywords:** Yak, Cattleyak, Epididymis, Sperm maturation, iTRAQ proteomics

## Abstract

**Background:**

During maturation, spermatozoa acquire motility and fertilizing capacity as they transit through the epididymis. In recent years, two-dimensional gel electrophoresis has been employed in proteomics studies conducted in rat, boar and human. However, there has not been a complete information regarding the proteins associated with sperm maturation in the epididymis. In this study, we employed iTRAQ proteomics to investigate proteins associated with sperm maturation between yak and cattleyak epididymis.

**Results:**

After a successful sampling and protein extraction, the iTRAQ coupled with LC-MS/MS mass spectrometry and bioinformatics analysis were performed. We identified 288 differentially abundant proteins (DAPs) between yak and cattleyak epididymis; 151 were up-regulated while 137 were down-regulated in cattleyak relative to yak. Gene Ontology analysis identified that down-regulated DAPs in cattleyak were mostly enriched in the acetylation of protein component, along with negative and positive regulatory activities. iTRAQ proteomics data showed that the top up-regulated DAPs were mainly enriched in cell communication, cell adhesion, cytoskeleton organization, stress response, post-translational modifications and metabolic functions while the down-regulated DAPs were predominantly associated with sperm maturation, long-term sperm storage, sperm forward motility, sperm-oocyte fusion and regulatory functions.

**Conclusion:**

These results provide insight into the molecular mechanisms underlying male cattleyak sterility.

## Background

Cattleyak is the hybrid of cattle (*Bos taurus*) and yak (*Bos grunniens*) that demonstrates strong adaptability to harsh environmental conditions in the adjacent Alpine regions and the Qinghai-Tibetan Plateau in China [1]. Cattleyak provides higher quantity of meat and milk as compared to cattle and yak. The male cattleyak is sterile and this may be associated with spermatogenic arrest [2, 3] and/or some other factors such as genes or proteins expression dynamics along the epididymis. The sterility of male cattleyak phenomenon poses a major challenge and has restricted the hybridization procedure for decades. In previous years, not too many proteomics studies have been conducted to investigate male cattleyak sterility [4, 5]. Most of the previous studies focused on testis and could not completely elucidate the exact cause of infertility in male cattleyak. Until now, no study has explored epididymis using iTRAQ proteomics as a means of identifying the sterility mechanism in male cattleyaks. Considering that testicular spermatozoa further undergo mandatory epididymal maturation in the epididymis, it is therefore a suitable organ for further investigating male cattleyak sterility.

The epididymis is a long complex convoluted tube found on the surface of each testis, which connects the efferent duct to the vas deferens in the male mammalian reproductive tract [6]. The epididymal epithelium consists of 5 main epithelial cell types: principal, clear, basal, narrow, and halo cells; which provide not only structural support to epididymis but also play significant role in the epididymal spermatozoa maturation process [7]. The epididymis is the site where newly produced testicular spermatozoa acquire the ability to swim, penetrate and complete fertilizing competency. These functions are carried out from the secretion by epididymal epithelial cells (EECs) of several key proteins to create an interactive and dynamic microenvironment in the lumen where the spermatozoa are stored, protected, and undergo maturation. Moreover, Skerget et al. (2015) reported that, during sperm passage through the epididymis, the sperm surface protein must be acquired, lost, and modified to confer motility and fertilization competency to sperms [8]. With their significant functions in the process of sperm maturation and male fertility, the proteins have received considerable attention and have been assumed to be prospective targets for identifying and treating infertility. However, until now, very limited knowledge is available about the exact roles of proteins in the regulation of the several important processes involved in the epididymal sperm maturation and fertilization.

Advances in bioinformatics have greatly helped in understanding of sperm proteome composition and function. In recent years, some proteome studies have been successfully conducted in rat, human, and boar [9–11] by employing two-dimensional gel electrophoresis. However, the gel-based studies may suffer from drawbacks such as representation of the protein types at low concentrations and nature of the protein such as acidic or basic proteins [5]. Isobaric tagging for relative and absolute protein quantification (iTRAQ) is a proteomics technique developed to quantitatively investigate protein abundance changes in different biological samples with high accuracy and reproducibility and the advantage of this multiplexing reagent is that 4 or 8 analysis samples can be quantified simultaneously [12, 13]. In this study, we identified proteins associated with sperm maturation between yak and cattleyak epididymis employing iTRAQ proteomics.

## Results

### Identification of differentially abundant proteins (DAPs) between yak and cattleyak epididymis by iTRAQ

**Fig. 1:**
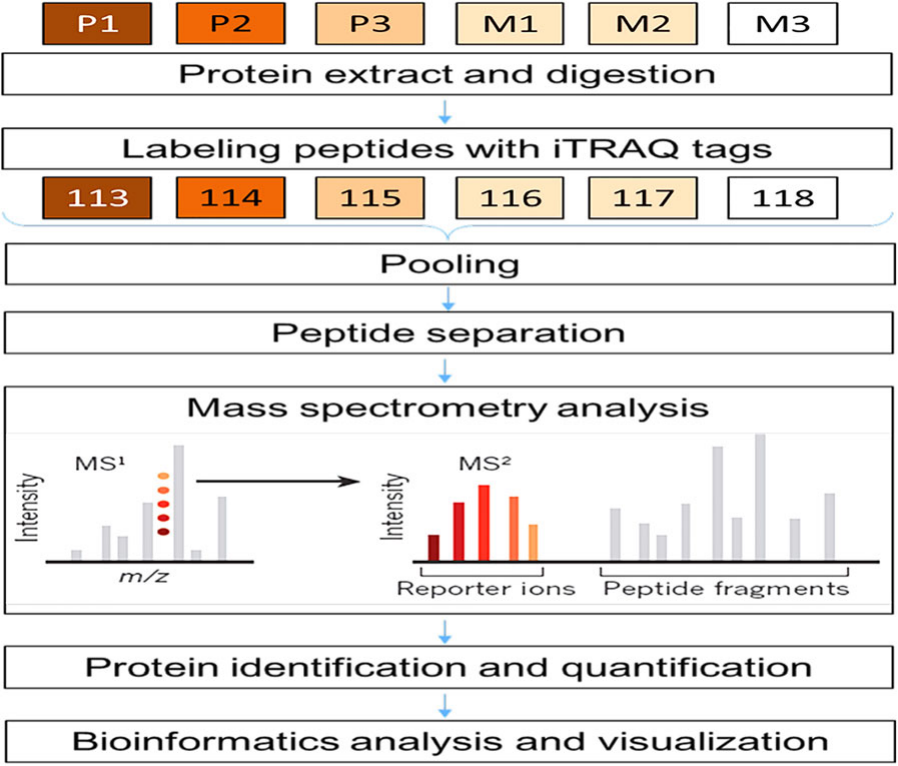
The schedules of experimental treatment for the comparison of yak and cattleyak epididymal proteome. Proteins extracted from epididymis tissue samples were diminished, alkylated and enzymatically digested before labeling with iTRAQ, followed by LC-MS/MS mass spectrometry analyses. In the last section, proteins were identified and quantified before analysing and visualization. Legends: Male cattleyaks (*n* = 3; age: 1 year; named P1, P2, and P3) and Male yaks (*n* = 3; age: 1 year; named M1, M2, and M3)’’

**Fig. 2:**
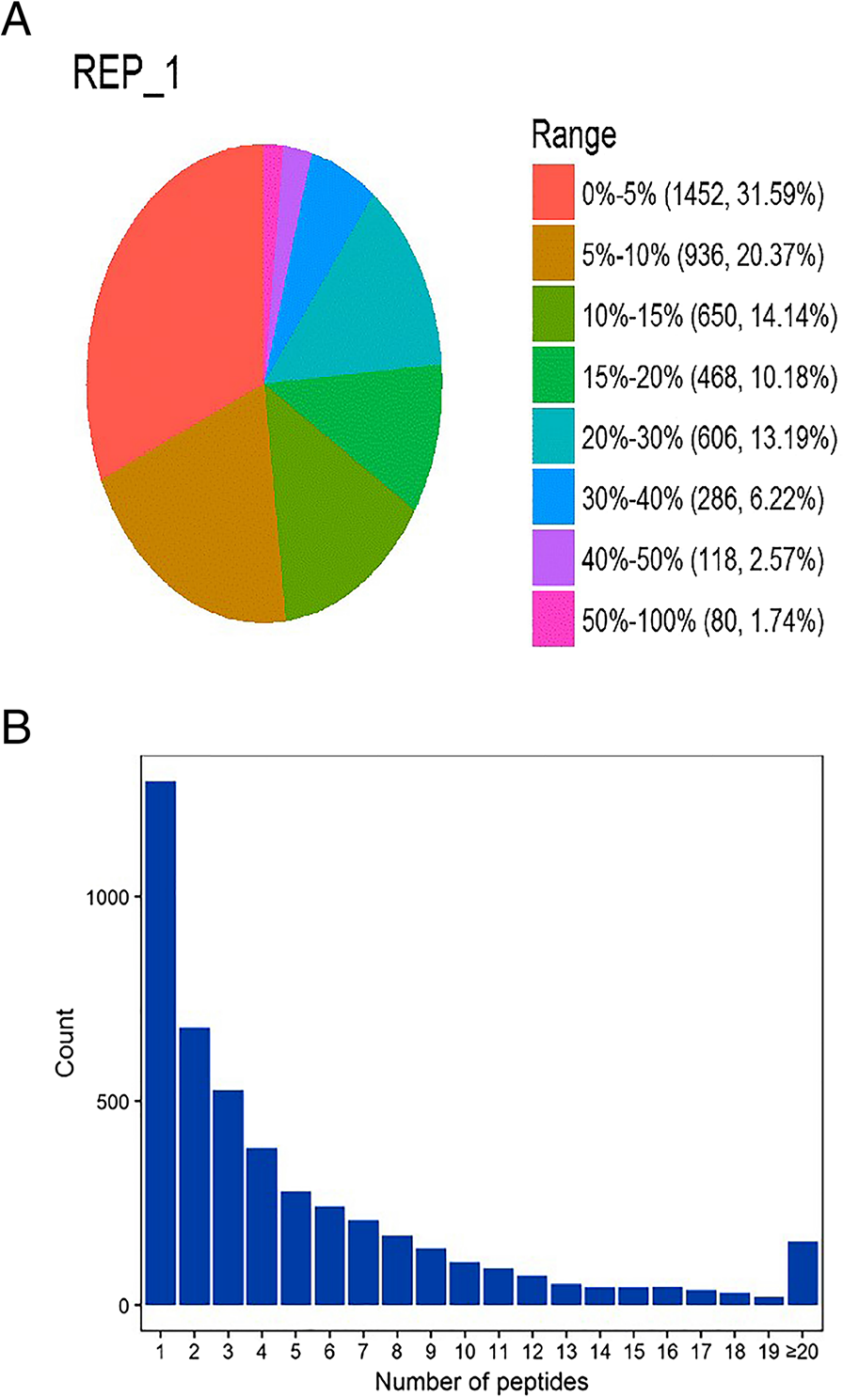
Peptide sequence coverage and identification of number of peptides. The **A** shows the proportion of proteins in different coverage ranges. The different colors representing different ranges of sequence coverage. The brackets show the number of proteins in different coverage ranges and their proportion to the total protein. The **B** shows the distribution of the number of peptides contained in the identified protein. The x-axis is the range of peptides covering the protein, and the y-axis is the number of proteins

We applied a quantitative iTRAQ-based proteomics approach for the determination of proteomic changes between yak and cattleyak epididymis. For the identification and relative quantification of proteins, an overview of workflow described by yak and cattleyak epididymis proteome comparison is shown in (Fig. 1). In 3 biological replicates, a total of 54,088 spectrums were generated and 4596 proteins were identified from 23,801 peptides between yak and cattleyak epididymis proteome. Normally, the number of proteins decreased from 1452 to 80, as the increase in the protein coverage ranges from 0 to 100% (Fig. 2A). For the identification of peptide segments distribution, the Fig. 2B shows that most of the identified proteins contain < 25 peptide segments and the number of proteins segments decreased as the number of matching peptide segments was increased.

### Quantification of DAPs between yak and cattleyak epididymis by iTRAQ

Out of the total identified proteins between yak and cattleyak epididymis proteome; 288 met the DAPs selection criteria, out of which 151 were up-regulated (fold change ≥1.5, *p* ≤ 0.05) and 137 were *p* ≤ 0.05). Down regulated DAPs including Vitamin D-binding protein, DnaJ homolog subfamily C member 17, and Serotransferrin had fold changes of 0.06, 0.08 and 0.10 respectively. On the other hand, 34 out of the 151 up-regulated proteins had fold change values ≥2; among these were uncharacterized protein, A-kinase anchor protein 3, Uncharacterized protein, MHC class I antigen (Fragment), and Beta A4 crystallin with fold changes of 4.6, 4.2, 3.9, 3.8, and 3.8 respectively. We have listed top 30 up-regulated DAPs and top 30 down-regulated DAPs identified from cattleyak relative to yak (Tables 1 and 2, respectively).

**Table 1:** The top 30 down-regulated differentially abundant proteins (DAPs) between yak and cattleyak epididymis

Accession	Description of Protein	Protein symbol	*P* value	FC (P/M)
F1N5M2	Vitamin D-binding protein	GC	4.65E-09	0.062
Q2KI83	DnaJ homolog subfamily C member 17	DNAJC17	0.000100554	0.081
G3X6N3	Serotransferrin	TF	0.006778114	0.106
A0JNP2	Secretoglobin family 1D member (LppAB)	SCGB1D	0.001735233	0.181
D4QBB3	Hemoglobin beta	HBB	1.06E-05	0.225
E1B9W6	Uncharacterized protein	ADCY10	0.000214338	0.241
A7Z033	T-complex protein 11-like protein 2	TCP11L2	0.007235083	0.256
P33433	Histidine-rich glycoprotein (Histidine-proline-rich glycoprotein) (HPRG) (Fragments)	HRG	7.60E-07	0.272
A5D7S6	PEF1 protein	PEF1	0.001584413	0.286
Q3MHV8	RBM15B protein (Fragment)	RBM15B	0.000115054	0.294
B0JYP6	IGK protein	IGK	0.002491825	0.295
G3N1U4	Serpin A3–3	SERPINA3–3	4.24E-07	0.306
Q9XSK2	CD63 antigen (CD antigen CD63)	CD63	7.98E-09	0.316
Q0IIG7	Ras-related protein Rab-5A	RAB5A	0.000214338	0.318
F6RF62	Uncharacterized protein	MYL4	1.97E-08	0.344
G9HQZ5	MHC class I antigen (Fragment)	BoLA	0.000559995	0.362
E1BND7	Uncharacterized protein	PPFIA2	0.007269613	0.373
Q58D67	Dynactin 4	DCTN4	2.36E-05	0.403
Q3SZK1	Angio-associated migratory cell protein	AAMP	0.004313819	0.409
E1BPI2	Non-specific serine/threonine protein kinase (EC 2.7.11.1)		0.006717303	0.412
Q3ZCH5	Zinc-alpha-2-glycoprotein (Zn-alpha-2-GP) (Zn-alpha-2-glycoprotein)	AZGP1	0.007264748	0.426
A6QQR0	WDR75 protein	WDR75	2.56E-05	0.427
Q2KJ61	Elongator complex protein 3 (EC 2.3.1.48)	ELP3	0.000855102	0.435
E1BIM7	Uncharacterized protein	CELF2	0.005137574	0.439
E1BGN3	Histone H3	HIST2H3D	2.16E-15	0.444
A1A4J3	Zinc finger, CCHC domain containing 3	ZCCHC3	0.000169658	0.449
E1BE33	Uncharacterized protein	ZEB2	1.58E-09	0.450
G3MZD8	Uncharacterized protein		0.002250709	0.452
E1BEP7	Uncharacterized protein	ELP2	0.00728467	0.459
Q08E58	Tubulin tyrosine ligase-like family, member 12	TTLL12	0.000147691	0.467

**Table 2:** The top 30 up- regulated differentially abundant proteins (DAPs) between yak and cattleyak epididymis

Accession	Description of Protein	Protein symbol	*P* value	FC (P/M)
E1BKY2	Uncharacterized protein		0.00881948	4.65
F1MJS8	A-kinase anchor protein 3	AKAP3	0.000526461	4.26
F1MIM1	Uncharacterized protein	LOC104976250	0.000889658	3.89
Q3YJL1	MHC class I antigen (Fragment)	BoLA	0.007469039	3.81
Q6DTZ8	Beta A4 crystallin (Beta-crystallin A4)	CRYBA4	8.45E-09	3.80
G3N2N9	Glutathione peroxidase	GPX5	7.28E-05	3.55
E1BE11	Uncharacterized protein	HMCN1	5.28E-09	3.53
F1MIM0	Uncharacterized protein		0.000618683	3.41
Q32KP8	Serine peptidase inhibitor-like, with Kunitz and WAP domains 1 (Eppin)	SPINLW1	1.30E-19	3.34
Q0VCG3	Parvalbumin alpha	PVALB	0.000112643	3.03
E1BGB7	Uncharacterized protein		1.07E-05	2.85
Q3MHJ9	Calcium/calmodulin-dependent protein kinase type II subunit beta (CaM kinase II subunit beta) (CaMK-II subunit beta)	CAMK2B	0.001572046	2.79
Q70IB2	Inactive ribonuclease-like protein 10 (Protein Train A)	RNASE10	0.000205134	2.77
F1MY32	Uncharacterized protein	LY6G5C	0.004856315	2.76
F1MSZ5	Uncharacterized protein	ADAM28	1.08E-09	2.72
P02192	Myoglobin	MB	3.62E-05	2.65
A6H742	Plastin-1	PLS1	7.90E-07	2.64
G3N2L2	Uncharacterized protein	RCN1	0.000161915	2.58
F1MTV5	Amino acid transporter	SLC1A5	0.004519988	2.56
F1N5W4	Uncharacterized protein	ENPP5	0.000276382	2.52
F1N2E1	Uncharacterized protein	WFDC8	0.0076419	2.45
Q2TBR5	Protein FAM166B	FAM166B	2.10E-08	2.42
Q0VCU3	Cathepsin F	CTSF	0.002189062	2.33
A7E340	Mucin 15, cell surface associated	MUC15	7.89E-33	2.32
E1BD73	Poly [ADP-ribose] polymerase (PARP) (EC 2.4.2.30)	PARP4	5.16E-14	2.31
F1MG20	17-beta-hydroxysteroid dehydrogenase type 6	HSD17B6	0.002590687	2.26
F1MV86	G1/S-specific cyclin-D3	CCND3	0.000102853	2.25
A6QQ08	SNCA protein (Fragment)	SNCA	0.009021835	2.24
F1MUC1	Uncharacterized protein	ABCC4	4.25E-06	2.21
E1BJ49	Uncharacterized protein	MASP2	0.000448626	2.18

### Gene ontology (GO) analysis of the differentially abundant proteins

**Fig. 3:**
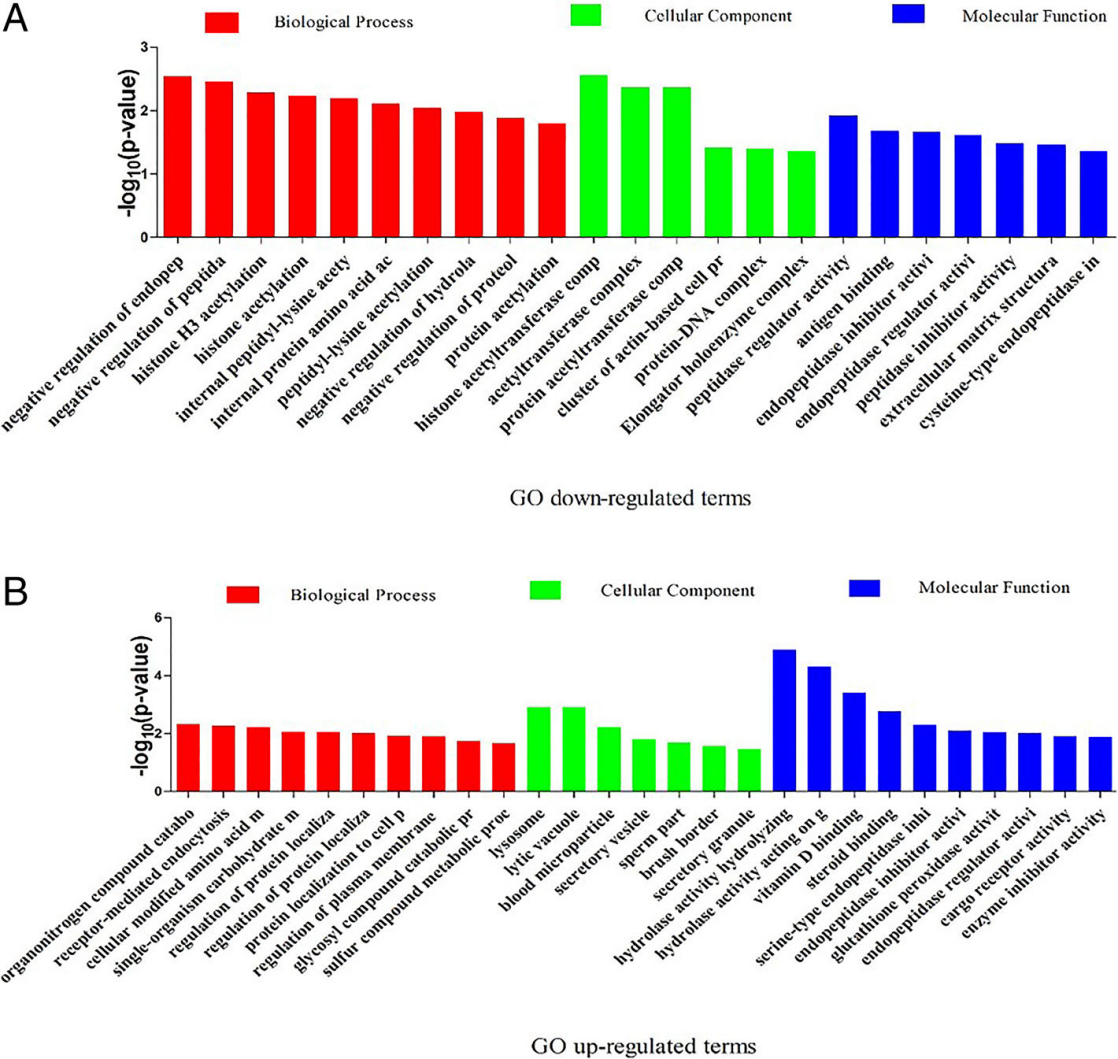
GO classification of DAPs in cattleyak compared to yak. The **A** describes GO terms of down-regulated DAPs and the **B** describes the GO terms of up-regulated DAPs. The x-axis represents the number of GO terms and y-axis represents *p*-value of each GO terms

Gene ontology (GO) analysis was conducted to better understand the functions of the all down-regulated and up-regulated DAPs identified by iTRAQ between yak and cattleyak based on biological process, cellular component, and molecular function categories. 35 GO terms were significantly enriched for down-regulated DAPs, including 22 biological process terms, 6 cellular component terms, and 7 molecular function terms. Majority of these down-regulated enriched GO terms relating to biological processes were mainly associated with acetylation of protein components, negative regulatory activity, and positive regulatory activity, from which top three listed enriched GO terms were negative regulation of endopeptidase activity (*p* = 0.002923), negative regulation of peptidase activity (*p* = 0.003519) and histone H3 acetylation (*p* = 0.005172). According to the down-regulated cellular components, most of enriched GO terms were associated with acetyltransferase complex, among which the top three listed enriched GO terms include histone acetyltransferase complex (*p* = 0.002818), acetyltransferase complex (*p* = 0.004352) and protein acetyltransferase complex (*p* = 0.004352). From down-regulated molecular functions, majority of enriched GO terms were involved in inhibitor activity and regulator activity, among which top three listed enriched GO terms include peptidase regulator activity (*p* = 0.011867), antigen binding (*p* = 0.020786), and endopeptidase inhibitor activity (*p* = 0.021568) (Fig. 3A), while, among the up-regulated DAPs, 48 GO terms were significantly enriched between yak and cattleyak epididymis, including 18 biological process terms, 7 cellular component terms, and 23 molecular function terms. Most of the up-regulated DAPs enriched GO terms in particular to biological processes were involved in metabolic processes, protein localization and catabolic processes, among which top three listed enriched GO terms include organonitrogen compound catabolic process (*p* = 0.004521), receptor-mediated endocytosis (*p* = 0.005261) and cellular modified amino acid metabolic process (*p* = 0.006143). With respect to cellular component, the most important up-regulated enriched GO terms were lysosome and sperm part, in which top three listed enriched GO terms were lysosome (*p* = 0.001189), lytic vacuole (*p* = 0.001189) and blood microparticle (*p* = 0.005987). Significantly up-regulated enriched GO terms based on molecular function were associated with binding, inhibitor activity, and hydrolase activity, in which top three enriched GO terms were hydrolase activity (*p* = 1.26E-05), vitamin D binding (*p* = 0.000388) and steroid binding (*p* = 0.001698) (Fig. 3B).

### KEGG pathway enrichment analysis of DAPs

**Fig. 4:**
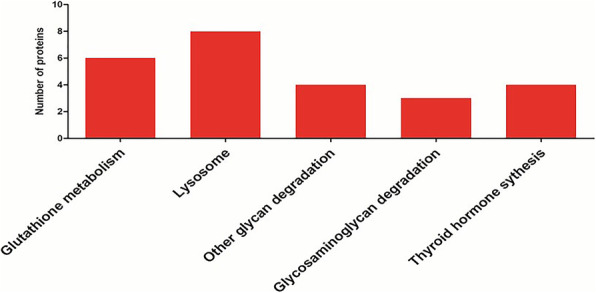
KEGG pathway enrichments analysis of DAPs in cattleyak with respect to yak. The x-axis displays each of the enriched pathways. The y-axis shows the number of DAPs in each pathway

Pathway analysis for identified proteins can deepen our understanding of the metabolic capacity of the species, biological processes information, and related diseases. In this study, we have mapped 87 down-regulated DAPs and 136 up-regulated DAPs to the reference pathways in the KEGG database to determine biological pathways related to sperm function. In total, five up-regulated DAPs were significantly enriched in KEGG pathways whereas none of the down-regulated DAPs were significantly enriched in KEGG pathways. For the up-regulated DAPs, the enriched KEGG pathways were associated with Glutathione metabolism (*p* = 9.75E-05), Lysosome (*p* = 0.000102), Glycan degradation (*p* = 0.000646), Glycosaminoglycan degradation (*p* = 0.01678) and Thyroid hormone synthesis (*p* = 0.021941) (Fig. 4).

### Protein-protein interaction (PPI) of DAPs

**Fig. 5:**
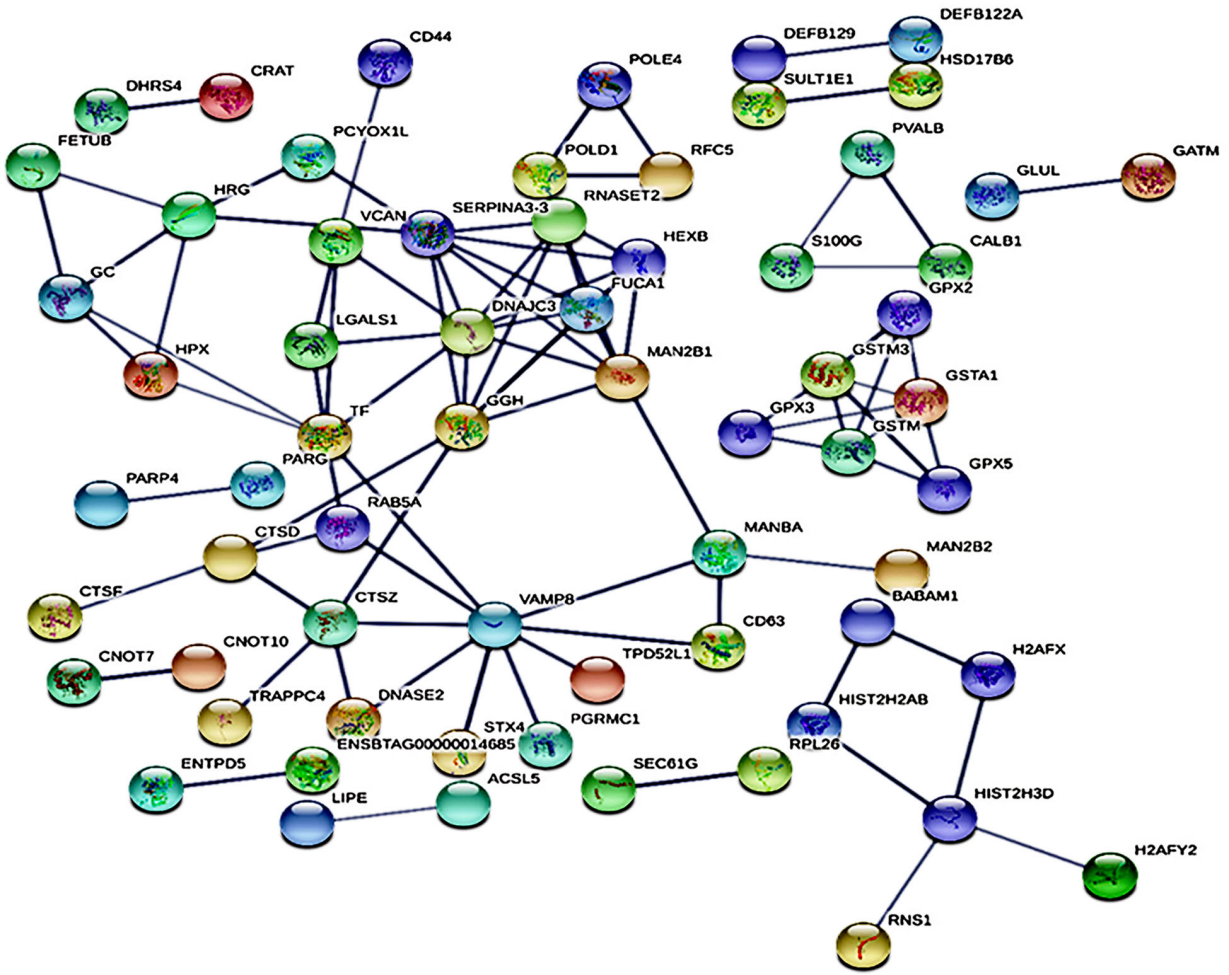
Protein-protein interaction (PPI) network of DAPs in cattleyak created by STRING database

**Fig. 6:**
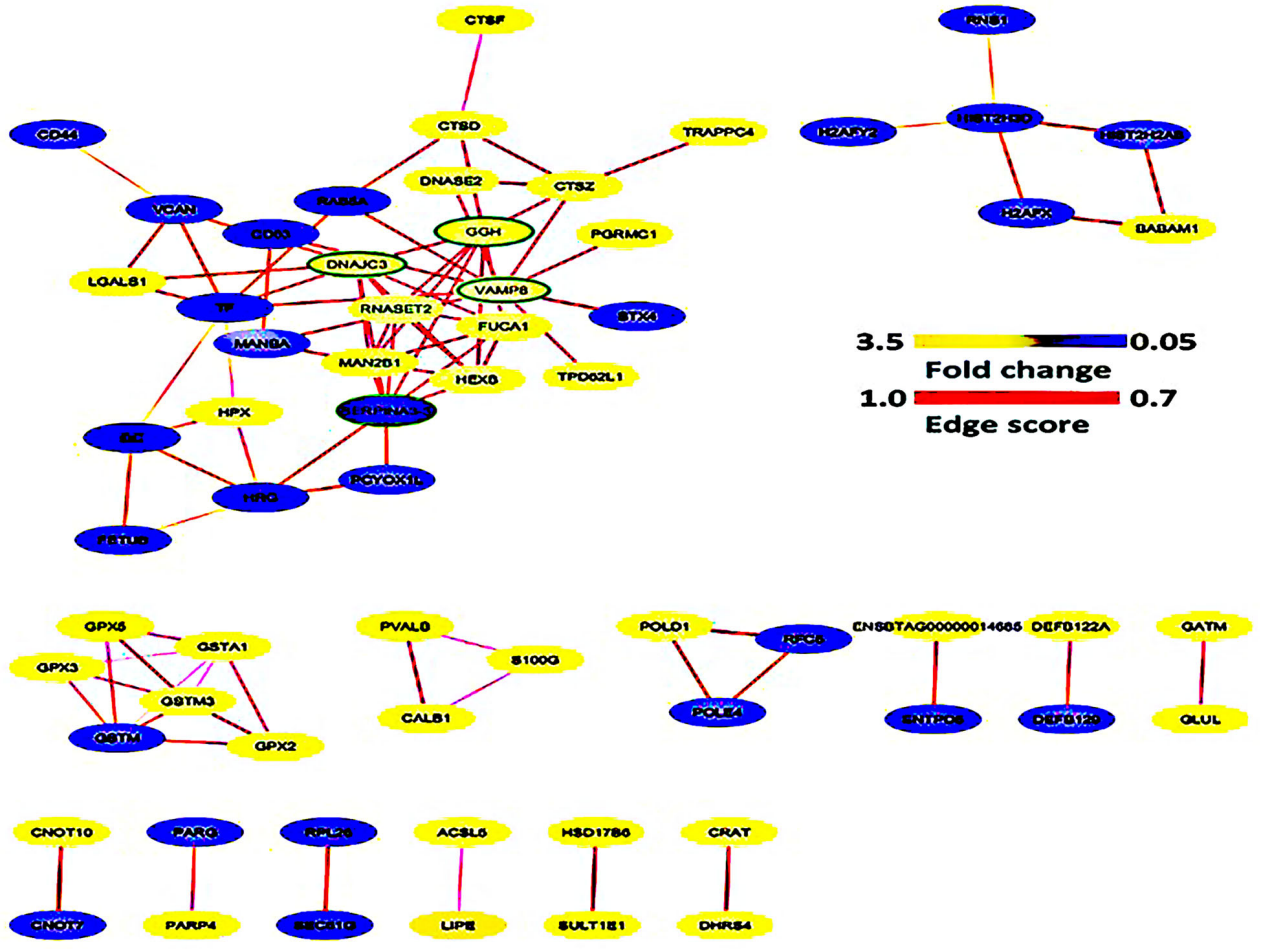
Protein-protein interaction (PPI) network of DAPs created with STRING database and visualized with Cytoscape. Proteins in blue were down-regulated and those in yellow were up-regulated. The line that connected the proteins with each other is called an edge. The color of the edges indicates the combined interaction score (edge score). The nodes with green borders described hub proteins

In this study, the Search Tool for the Retrieval of Interacting Genes /Proteins 11.0 (STRING 11.0) database was used for identifying protein-protein interaction network of DAPs. After removing unconnected and self-loops proteins, the resulting PPI network generated 64 protein nodes and 90 edges (in Fig. 5). Furthermore, the network data file was directly imported into Cytoscape software for visual editing with a threshold value of a combined score > 0.7. DAPs that connected higher than others in created network were considered as hub proteins (Fig. 6); these hub proteins may have significant roles in the regulation of the network. In the PPI network, the top four hub proteins include vesicle-associated membrane protein 8 (VAMP8), SERPINA3–3, GGH, and DNAJC3.

### Elisa

**Fig. 7:**
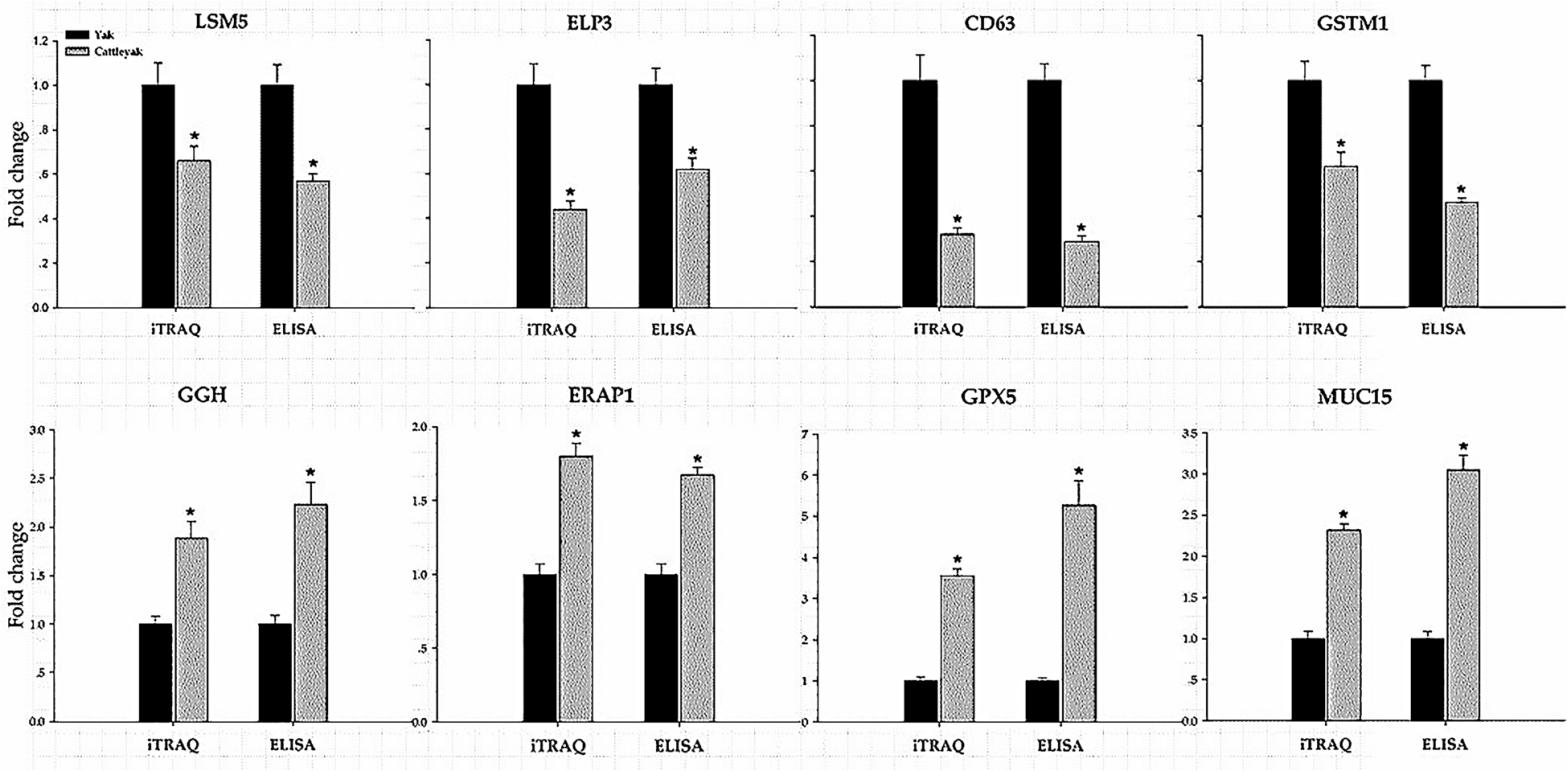
Validation of iTRAQ data with ELISA. The vertical axis indicates the fold change of protein abundance. The horizontal axis indicates the methods for ELISA and iTRAQ. In each method, the protein expression level of yak was the control and designated as 1 to determine the expression level of cattleyak. Expression level of each protein is the mean of three replicates for cattleyak and plotted (mean ± SD) in relation to yak as control (Y = 1). The expression pattern of the protein CD63, ELP3, LSM5, GSTM1, GGH, ERAP1, GPX5 and MUC15 was validated respectively. *Protein expression were significantly different from control (*p* < 0.05, Student’s t-test)

ELISA was performed to verify the results obtained from iTRAQ proteomics. The up-regulation of GGH, RAP1, GPX5 and MUC15 in cattleyak with respect to yak determined by iTRAQ was confirmed by ELISA (*p* < 0.05). Meanwhile, ELISA also verified the decreased expression of CD63, ELP3, LSM5 and GSTM1 in cattleyak compared to yak as identified by iTRAQ (*p* < 0.05) (Fig. 7). All these data validated the results obtained from iTRAQ.

## Discussion

Proteomics technologies present major possibilities to investigate the molecular mechanisms that regulate the functional activities of the sperm [14]. Initially, the iTRAQ proteomics technique was used to study male cattleyak infertility with an increased focus on the testis. Until now, no research has used the aforementioned methodology to analyze the epididymis, which is recognized to be morphologically and functionally active in sperm maturation and fertility. In this study, iTRAQ proteomics is employed in the analysis of proteins associated with sperm maturation in the epididymis of yak and cattleyak. The epididymis serves important functions, including post-testicular sperm maturation which is important as it provides suitable environment for spermatozoa to develop progressive movement capacity and fertilization competence. The dynamic nature of the epididymal environment ensures that problems in the signaling pathways lead to infertility. In the epididymis the sperm protein, lipid and small RNA content are highly modified as a result of the continuous interaction with luminal proteins mainly secreted by the epididymal epithelium and extracellular vesicles, epididymosome [15]. Epididymal maturation happens as structural modifications in the epididymis occur, allowing sperm to capacitate in the female reproductive tract [16]. This study focuses on epididymal proteins and shows how they affect sperm functions. However, there exist major group of proteins identified to be expressed in testis as well as epididymis and they play specific important roles in sperm functions.

### Epididymal sperm maturation

The functional analysis identified a number of down-regulated DAPs involved in epididymal maturation in cattleyak (Table 1). Phosphatidylethanolamine-binding protein 4 (PEBP4) was studied in fertile, low fertile, and infertile bulls and found that the novel seminal PEBP4 expression was significantly higher in fertile bulls relative to low fertile and infertile bulls, implying that PEBP4 could play important roles in spermiogenesis, epididymal sperm maturation, and sperm motility [17]. The existing evidence suggests that, down-regulation of PEBP4 in cattleyak could compromise sperm maturation processes and result in infertility. CD63, is a recognized exosome marker that is found in the apices of epididymal epithelial principal cells and the epididymal microenvironment, allowing for long-term sperm storage [18]. The protein facilitates signal transduction functions, which are important in regulating cellular proliferation, growth, and motility. Downregulation of CD63 in cattleyak could have a significant impact on sperm storage and lead to fertility problems. Seminal plasma contains proteins associated with sperm progressive motility, including Zinc-alpha-2-glycoprotein (ZAG) and could play functional roles during maturation of spermatozoa, from the epididymis through fertilization in the female reproductive tract [19]. The downregulation of ZAG in cattleyak suggests that sperms may well not develop progressive motility after ejaculation through into female reproductive tract.

Glutathione S-transferase Mu 1 (GSTM1–1) and Fetuin-B were also found to be down-regulated in cattleyak compared to yak. The possible role of Glutathione S-transferase Mu 1 (GSTM1–1) in the epididymis is unspecified, despite the fact that it is a member of the Glutathione S-transferase mu class (GSTM1) found on sperms. GSTM1 is known to be associated with sperm-oocyte fusion and promotes precise joining of sperm surface-active components with the zona pellucida (ZP) during the sperm-egg fusion process [20, 21]. Decreased GSTM1–1 expression could be a potential cause of reduced or impaired fertilization in cattleyak. Fetuin-B was identified in mice as a plasma protein inhibiting ovastacin, a cortical granula protease known to cause ZP hardening and infertility [22, 23]. It is possible that downregulation of Fetuin-B in cattleyak resulted in similar effects. The biological roles of the aforementioned proteins indicate that their downregulation could have a detrimental impact on sperm maturation, long-term sperm storage, sperm forward motility, and sperm-oocyte interaction.

### Epididymal dysfunction

In general, the epididymis comprises a variety of molecules and proteins that aid sperms in their quest to fertilize oocytes. Nuclear pores, which are essential in the exchange of macromolecules such as proteins and RNAs, facilitate interaction between the nucleus and the cytoplasm in eukaryotic cells. The nuclear localization signal (NLS), which is made up of 100 different proteins, particularly Importin subunit alpha, mediates active nuclear protein transport by recognizing karyophile proteins and forming a stable complex known as the nuclear pore-targeting complex (PTAC) [24, 25]. Importin subunit alpha’s biological activity indicates that increased expression of this protein may block the critical contact between nucleus and cytoplasm in cattleyak epididymal cells, causing infertility. Mucins form highly glycosylated proteins which are classified into two types: secretory mucins and membrane-bound mucins. Mucin 15 have been discovered to be a membrane-bound mucin expressed by epithelial cells, implying that changes in membrane-bound mucins could affect cellular development, differentiation, transformation, invasion, and adhesion [26]. Mucin 15 upregulation in cattleyak suggests that Mucin 15 could perhaps enhance adhesion, which may affect or interfere with sperm cell migration through the epididymis. GAS2 was discovered to be a part of the microfilament system, associated with cell growth arrest through a phosphorylation mechanism during the Go-G1 transition [27]. GAS2 overexpression causes membrane ruffling and the termination of mitosis [28]. The upregulation of GAS2 in cattleyak could have affected the vital molecules or cells participating in the overall growth of sperms in the cattleyak epididymis.

Aminopeptidase and Poly (ADP-ribose) polymerase (PARP) were also found to be up-regulated in cattleyak and are known to be involved in a variety of stress responses. Aminopeptidase, an enzyme encoded by the Endoplasmic reticulum aminopeptidase 1 (ERAP1) gene, was thus discovered to play a key role in immune response [29]. Aminopeptidase perform functions similar to acute-phase proteins thus, it is secreted into the blood in response to inflammatory stimuli and is involved in increasing nitric oxide (NO) synthesis as a host defense mechanism [30]. Poly (ADP-ribose) polymerase (PARP) functions as a DNA repair enzyme present in a variety of tissues, including the epididymis and it is activated when a DNA strand splits as a result of reactive oxygen species (ROS) or oxidative stress [31, 32]. PARP is activated by nicotinamide-adenosine dinucleotide (NAD) which results in ADP-ribosylation of PARP. Excessive PARP activation has been attributed to NAD+ depletion and corresponding cellular ATP depletion, eventually leading to necrotic-type cell death [33]. This could be explained that increased Aminopeptidase and PARP expression levels led to an increase in a constant stress state of sperm cells involving the acquisition of certain toxic substances formed by altered DAPs. Moreover, mammalian epididymal spermatozoa achieve fertility development by a mechanism known as post-translational modifications [34]. Polypeptide N-acetylgalactosaminyltransferase 6 (GalNAc-T6) is a member of the polypeptide N-acetylgalactosaminyltransferase (GalNAc-Ts) family that take active part in catalyzing the transport of N-acetyl—D-galactosamine: polypeptide (GalNAc) from the sugar donor uridine diphosphate (UDP)-GalNAc to the serine and threonine residues of glycoproteins [35]. Previous research has shown that the modulation of spermatozoa plasma glycoproteins is essential for the development of functionally mature sperms during migration through the epididymis [36, 37]. As a result, up-regulation of GalNAc-T6 could modulate glycosylation and have a significant negative effect on sperm motility and perhaps on the fertility process.

Proteolytic cleavages of surface proteins by protease enzymes are essential for the interactions of spermatozoa and zona pellucida. Protease inhibitors regulating the sperm membranes prevent premature capacitation, thereby controlling the proteolytic cascades [38]. Serpin A3–8 was discovered in buffalos as a protease inhibitor and served active in the protease inhibition mechanism [39]. An essential Serine peptidase inhibitor-like protein (M/*P* = 3.339198) with Kunitz and WAP domains 1 (Eppin) were discovered in male monkeys and the high-titer monkeys were found to be infertile [40]. As a result of the increased expression of these protease inhibitors, they could inhibit proteolytic cleavages needed for the early events of fertilization. Consequently, the biological functions of the proteins were associated with cell communication, cell adhesion, cytoskeleton organization, stresses, and post-translational modifications.

### Regulatory mechanisms

Different luminal environments exist within the epididymis, an undifferentiated epithelium undergo various sequence of modifications to become completely differentiated, with differences in anatomy, gene regulating activities, and functions [41]. The mechanisms involved in gene expression are regulated at many different levels, from transcription to protein post-translational modifications. These highly complex processes necessitate the involvement of a variety of key proteins that are responsible for the activation of critical pathways. GO biological function analyses revealed a number of down-regulated DAPs involved in regulation and acetylation. The central objective of control in gene expression mechanisms is the transcriptional level, where histones and their acetylation in nucleosomes play functional roles in regulating gene expression. Histone H2A and its variant Core histone macro-H2A were identified as members of histone octamer that formed a structural unit (nucleosome) of chromatin [42, 43]. Histone H2A possesses N-terminal tail (H2ANtT) and C-terminal tail (H2ACtT). The H2ANtT perform essential functions mainly in inter-nucleosome interaction [44], while H2ACtT recruits linker histone H1 to the nucleosome and stabilizes it [45]. Deletion of H2ACtT could increase H2A mobility and also decrease nucleosome stability. Elongator complex protein 3 (ELP3) was found as a catalytic subunit of Elongator protein encoding a histone acetyltransferase and facilitating histone acetylation [46]. Acetylation of histones permits chromatin to reveal DNA binding sites and initiate transcription [47]. As a result, it is possible that the down-regulation of Histone H2A and ELP3 in cattleyak is a source of transcriptionally silenced genes that encode essential epididymal sperm maturational proteins.

Post-transcriptional regulation of genes could be controlled by the processes of deadenylation and pre-mRNA splicing. CCR4-NOT transcription complex subunit 7 or CCR4-associated factor 1 (CAF-1) serves as a deadenylase enzyme in the mRNA degradation process, during which poly (A) tail is eliminated and the newly synthesized mRNA is highly decreased from 200 to 250 nucleotides to 10–60 nucleotides [48, 49]. The poly (A) tail length is considered important in mRNA stability, translation efficiency, and gene regulation [50], whereas U6 snRNA-associated Sm-like protein LSm5 serves as a major component of spliceosome initiating significant functions in pre-mRNA splicing mechanisms, resulting in the development of maturated mRNA [51]. Multiple protein isoforms could be derived from a single gene during the pre-mRNA splicing process, and they could play an important role in cellular differentiation and organism growth [52]. Down-regulation of U6 snRNA-associated Sm-like protein LSm5 could facilitate the disruption of normal cellular functions, resulting in infertility. Furthermore, Aspartyl-tRNA synthetase (AspRS) and Protein transport protein Sec61 subunit gamma were discovered to be active in translational and post-translational processes, respectively. Aminoacylation is the mechanism by which tRNAs are charged prior to initiating the translation process. AspRS is an aminoacyl-tRNA synthetase which charges its cognate tRNA with aspartate amino acid and is involved in the oxidative stress response [53]. However, transport protein Sec61 subunit gamma which is a component of protein-conducting channel (PCC) resides in the membrane of the endoplasmic reticulum, where it binds to translating ribosomes for co-translational protein transport [54]. The biological functions of the translational and post-translational proteins revealed that their down-regulation may result in the inability of proteins to fold efficiently in the endoplasmic reticulum (ER).

### Metabolic functions

Metabolism involves series of chemical and physical modifications that develop in the body of animals, providing energy for the synthesis of newly required life processes. GO biological function analysis revealed a number of up-regulated DAPs involved in certain metabolism processes in cattleyak. It was previously reported that targeting sperm and energy metabolism have a stronger relationship [55]. We recognized specific significant candidates for up-regulated DAPs involved in metabolic functions in this study: Biliverdin reductase A (BVR A) and Gamma-glutamyl hydrolase (GGH). In mammalian epididymis, Biliverdin (BV) and its reduced form, bilirubin (BR), perform essential functions. BVRA was identified as an enzyme that converted biliverdin to bilirubin and therefore considered to play a crucial role in scavenging reactive oxygen species (ROS), reactive nitrogen species (RNS), and nitric oxide through its substrate (BV), which is also a by-product (BR) [56, 57]. The increased expression of BVRA in the epididymis of cattleyaks could restrict the capacity and quantity of biliverdin to facilitate certain oxidant activities. Proteins, micro and macronutrients all play important roles in fertilization; folate is a micronutrient that remains essential for sperm quality. Sperm aneuploidy has a negative impact on sperm production and could be reduced by consuming 400 mg to 700 mg total folate per day [58]. Gamma-glutamyl hydrolase (GGH) was discovered to be a lysosomal enzyme active in dietary folylpoly-y-glutamate metabolism, allowing folylmonoglutamate to reach the intestinal membrane to play a significant functions in folate cellular homeostasis [59]. The biological function of GGH suggests that overexpression of this protein could compromise cellular folate maintenance and have a negative impact on cattleyak sperm production.

## Conclusion

We identified a total of 4596 proteins between yak and cattleyak epididymis. Out of the total, 288 were DAPs, of which 151 were up-regulated DAPs and 137 were down-regulated DAPs. Most of the identified proteins in cattleyak were involved in epididymal sperm maturation, epididymal dysfunction, regulatory and metabolic functions. iTRAQ proteomics data identified several key DAPs (PEBP4, CD63, ZAG, GSTM1–1, and Fetuin-B) that potentially play significant roles in the sperm maturation process in cattleyak. These results provide insight into the molecular mechanisms underlying male cattleyak sterility as upregulation and downregulation of certain important DAPs in the epididymis impede the normal development of a fertile ejaculate and represent a part of the causes of infertility. Continuously understanding the specific functional descriptions of the key DAPs may facilitate proper breeding techniques. Numerous DAPs from this study may be useful for further studies on the molecular mechanisms causing male cattleyak sterility.

## Methods

### Animals and epididymis sample collection

Samples were collected under license in compliance with the Chinese Guidelines for the Care and Use of Laboratory Animals, and all procedures were authorized by the Southwest University of Science and Technology’s Institutional Review Board [60]. Male yaks (Maiwa yaks) (*n* = 3; age: 1 year; named M1, M2, and M3) and Male cattleyaks (Maiwa yak × Tibetan taurine) (*n* = 3; age: 1 year; named P1, P2, and P3) were sampled from Maiwa yak population fed on a farm in Hongyuan county, Sichuan province of China.

Veterinary surgery was used to extract epididymides from yaks and cattleyaks, fat and connective tissues were removed. Epididymides were separated apart from testis by fine-scale dissection and preserved in liquid nitrogen (− 196 °C), transported to the laboratory and stored at − 80 °C until further analysis. After the samples were collected, the animals were treated and stored in a safe and enclosed area near to the farm-house.

### Total protein extraction from epididymal tissue

Total protein was extracted from the epididymal tissue that contained sperm. Cryogenic grinding with mortar and pestle of each epididymal tissue sample was performed and for protein stability, lysis buffer (7 M urea, 2 M thiourea, 0.1% CHAPS) was added and vortexed to mix. Ultrasonic extraction of lysates, centrifugation and storage followed [61]. The spectroscopic Bradford protein assay was used to measure the concentration of the extracted protein. Sample dilution in lysis buffer was performed for the final concentration within the range of the standard curve. BSA was dissolved with lysis buffer to a series of standard protein concentrations. Separate diluted sample 10 μL (standard) reacted respectively with 300 μL protein quantitation dye for 15 ~ 20 min. Simultaneously, absorbance was measured and sample concentrations were calculated based on the standard curve [5].

### Enzymatic digestion of the proteins

100 μg of total protein sample was used by a filter-aided sample preparation (FASP) protocol. Addition of 10 μL of reducing reagent to the samples at 37 °C for 1 h and 2 μL of cysteine-blocking reagent for 30 min at room temperature followed. The reductive alkylated protein solution was then added to a 10 K ultrafiltration tube (Merck Millipore, REF UFC501096), centrifuged at 12,000 rpm for 20 min, and bottom solution of the collection tube was discarded. Afterwards, 100 μL dissolution buffer was added in the iTRAQ kit and centrifuged at 12,000 rpm for 20 min. Prior to collection tube replacement, the settled solution in the collection tube was discarded, and there were three repetitions. Subsequently, 4 μg (1:50 compared to protein) trypsin (Promega REF V5111) in a volume of 100 μL was added and waited to react [5]. Sample centrifugation at 12,000 rpm for 15 min was carried out on the following day. Subsequent to enzymatic digestion, the peptide solution was retained at the bottom of the collection tube. Ultrafiltration tube was used with 200 μL of ddH2O and centrifugation at 12,000 rpm for 15 min followed. Resulting 500 μL of the digested sample was collected from the collection tube.

### iTRAQ labeling

iTRAQ reagent (8-plex) was set to reach room temperature. 150 μL of isopropanol was added to each tube of iTRAQ reagent and vortexed to mix before centrifuged. A new centrifuge with 50 μL of sample (100 μg of enzymatic product) was transferred, iTRAQ reagent was added, vortexed and centrifuged. pH testing followed with 0.5 μL of the solution on pH paper of pH range 7.0 to 10.0. The tubes were then incubated at room temperature for 2 h. To stop the reaction, 100 μL of ddH2O was added to the mixture.

### Offline pre-separation of enzymatically digested peptides and LC-MS/MS mass spectrometry

After dissolving the labelled lyophilized peptides [5], the samples were centrifuged at 14,000 rpm for 10 min, and the supernatant was collected for use. Assessing the isolation conditions followed and 100 μL of the total prepared sample was loaded at a flow rate of 0.7 mL/mins and separation gradient were used [61]. Protein analysis and the nanoscale reversed-phase chromatography was facilitated by mass spectrometer (Thermo, Model: Fusion). The high pH reversed-phase resulting segments were reconstituted with 20 μL (2% methanol, Sigma-Aldrich, article number: 14262, USA), (0.1% formic acid, Sigma-Aldrich, Cat. No. 56302, USA), and 13,000 rpm centrifugation for 10 min followed. Loading by sandwich technique, isolation flow along with isolation gradients followed [60]. The mass spectroscopy (MS) parameter was set as: spray voltage 2.1 kV; capillary temperature: 250 °C, and Scan range: 350-1800 m/z.

### Data analysis

Bovine reference genome (ARS-UCD1.2) was used, and Fusion mass spectrometry was used for mass spectrometry analysis of iTRAQ. The original mass spectrometry data were processed uisng Thermo’s commercial software Proteome Discoverer 1.4. Search parameters were used and Mascot facilitated an automated decoy database search by selecting the decoy checkbox [5]. The dataset collected proteins that existed in all three biological replicates. The observed findings were based on 95% percent confidence intervals for protein recognition as calculated by a Mascot probability study. Proteins with confidence intervals greater than “identity” were considered to be known. At least one distinct peptide aided in protein recognition. Protein quantification was determined by proteins with at least two distinct spectra.

For protein quantification, it was required that a protein contains at least two unique spectra. MASCOT was used for Quantification protein ratios. The significant differentially abundant proteins (DAPs) were screened by T-test and proteins with a *p*-value less than 0.05 and a fold difference greater than 1.5 (Up regulated) or less than 0.67 (Down regulated) were considered DAPs.

### GO, KEGG and PPI analysis of DAPs

Gene Ontology (GO) terms in the database (http://www.geneontology.org/) were used for mapping of the DAPs between yak and cattleyak epididymal tissues. Bonferroni Correction was used to normally adjust the *p*-value. Enrichment of biological pathways of the DAPs utilised KEGG database (Kyoto Encyclopedia of Genes and Genomes database). String Protein Interaction Database was used and the differential protein interaction network data files were complemented with Cytoscape software for visual editing.

### Enzyme linked immunosorbent assay (ELISA)

ELISA confirmed eight differential abundance proteins, namely CD63, ELP3, LSM5, GSTM1, GGH, ERAP1, GPX5 and MUC15**.** The total protein of each sample was extracted according to the manufacture’s protocol of DNA/RNA/protein co-extraction Kit (Tiangen Biotech (Beijing) Co., Ltd., China). The different sample protein concentration was detected by NanoDrop 3000 Spectrophotometer (Thermo Fisher Scientific, Wilmington, DE, USA) and adjusted to 0.1 mg/mL. ELISA detection followed [5] and standard curve as well as regression equation were made using the manufacturer’s standard samples with each sample analyzed with triplicates. Each well’s OD value detection was noted using microplate reader, set to 450 nm within 15 min. Using the regression equation, the targeted protein concentrations were calculated and each protein content was analyzed from each sample. Statistical analyses were performed using SPSS 22.0 standard version (IBM, Armonk, NY, USA). Student’s t-test was employed to analyze differences of each protein expression level between the control (yak) and tested groups (cattleyak). For all tests, statistical significance was taken as *p* < 0.05.

## Data Availability

All data included in this study are available upon request by contact with the corresponding author.

## Abbreviations

AspRS: Aspartyl-tRNA synthetase
BVR A: Biliverdin reductase A
CAF-1: CCR4-associated factor 1
DAPs: Differentially expressed proteins
ddH2O: Double distilled water
EECs: Epididymal epithelial cells
ELISA: Enzyme linked immunosorbent assay
ELP3: Elongator complex protein 3
ERAP1: Endoplasmic reticulum aminopeptidase 1
FASP: Filter-aided sample preparation
GalNAc-T6: N-acetylgalactosaminyltransferase 6
GGH: Gamma-glutamyl hydrolase
GO: Gene ontology
GPX5: Glutathione Peroxidase 5
GSTM1: Glutathione S-transferase
iTRAQ: Isobaric tags for relative and absolute quantitation
KEGG Database: Kyoto Encyclopedia of Genes and Genomes database
MUC15: Mucin 15
NLS: Nuclear localization signal
PARP: Poly (ADP-ribose) polymerase
PCC: Protein-conducting channel
PEBP4: Phosphatidylethanolamine-binding protein 4
PTAC: Pore-targeting complex
RAP1: Ras- Proximate-1/ Ras- related protein 1
RNS: Reactive nitrogen species
ROS: Reactive oxygen species
SERPINA3: Serpin family A member 3
VAMP8: Vesicle-associated membrane protein 8
ZAG: Zinc-alpha-2-glycoprotein
